# Global urban homogenization and the loss of emotions

**DOI:** 10.1038/s41598-022-27141-7

**Published:** 2022-12-29

**Authors:** Adrienne Grêt-Regamey, Marcelo Galleguillos-Torres

**Affiliations:** Planning of Landscape and Urban Systems, Institute for Spatial and Landscape Development, ETH Zürich, Zürich, Switzerland

**Keywords:** Psychology and behaviour, Environmental impact

## Abstract

Urban expansion is generating unprecedented homogenization of landscapes across the world. This uniformization of urban forms brings along dramatic environmental, social, and health problems. Reverting such processes requires activating people’s sense of place, their feeling of caring for their surroundings, and their community engagement. While emotions are known to have a modulating effect on behavior, their role in urban transformation is unknown. Drawing on large cognitive-psychological experiments in two countries, we demonstrate for the first time that urban homogenization processes lower people’s affective bounds to places and ultimately their intentions to engage with their neighbourhoods. The dulled emotional responses in peri-urban areas compared to urban and rural areas can be explained by lower social cohesion and place attachment. The findings highlight the significance of considering emotions in shaping just, equitable, sustainable, and resilient cities.

## Introduction

Across the world, urban areas are growing much faster than the urban population^1,2^. This pervasive expansion takes places where urban and rural land uses closely blend and form a new type of landscape, which is neither rural nor urban, often called the peri-urban area^3,4^. While this peri-urbanization process takes place in highly different contexts, it generates similar urban patterns across the world^5,6^. Standardized business centers, single-family residential areas and shopping malls displace highly productive agricultural land, eliminating the distinct and recognizable pattern of elements that makes one landscape different from another. Such homogenization of urban forms are known to lower the tie between people and place^7^, and their sense of place^8^. Breaking the dynamics of such processes would require fostering people’s feelings of caring for the local community^9,10^, and ultimately their engagement in civic activities.

Emotions have been shown to modulate human decision-making^11–13^. The feeling of attachment to a place, for example, has been identified as an essential factor affecting pro-environmental behaviors^14,15^. Behavioral responses depend, however, not only on the conscious but also on the physiological emotional responses^16,17^. Unconscious attitudes have been shown to influence preferences^18^, social behavior^19^, or consummatory decision-making^20^. Until now, research in urban sciences has mostly harnessed cognitive and revealed behavioral measures rather than physiological measures to investigate people–place relationships^21–23^. Building on the seminal work of Ulrich^24^, studies have shown that stressful environments^25^ or the type of neighborhoods influence physiological reactivity^26–30^. In particular, exposure to urban green^26,31–35^ and high biodiversity^36–38^ have been shown to reduce stress, and natural sounds are more effective than traffic noise in fostering restorative experience^39^. The nature and role of bodily responses in the intentions to interact with places remains unclear.

Measuring emotions in real-world contexts is highly challenging, as one cannot control exposures across individuals. Virtual reality experiments are able to achieve a high level of experimental control^40,41^, and deliver experience that gives rise to illusory sense of presence^42,43^. They are particularly useful for visualizing geographic locations^44^, and have been useful to investigate stress in various environments^25,26,45,46^. Immersive projection is particularly important to investigate bodily responses to environmental stimuli^47^. The potential for experimental control and systematic variations makes them suitable to be linked to physiological measures^44,48,49^. Skin conductance, for example, has been used as a biomarker of unconscious arousal with well-known psychophysiological functioning^50–52^. It refers to changes in the electrical potential of the skin and signals activation of the sympathetic autonomic nervous system^52,53^. Complemented with self-reported scales^54,55^, which rely on individual awareness of emotional reactions, these techniques provide great opportunities for measuring both unconscious and conscious arousal in controlled environmental settings.

In this study, we harness the potential of virtual reality experiments and physiological measures to investigate if homogenized residential peri-urban landscapes trigger different emotional responses when compared to more urban or rural landscapes. We collect both physiological and cognitive responses to various landscapes scenes and explore the reasons that could explain people’s affective bonds to places. We focus on place-making, as the process by which meaningful places emerge out of various interactions between people and their environments^56–58^. A better understanding of place-making offers the potential of engaging the so-called deep leverage points for sustainable transformations^59–61^. We divide the skin conductance signal, termed electrodermal activity (EDA), into a slowly and continuously changing tonic skin conductance level and the faster-varying phasic component, both serving as valid indicators for arousal^52,62^. We complement the physiological measures of emotion with the non-verbal pictorial Self-Assessment Manikin (SAM) technique^55^ to assess the conscious arousal and valence (pleasure). Finally, using a questionnaire, we collect information on individuals’ perceived experience and affective bonds with places using well-studied concepts such as place attachment (Bonaiuto et al., 2015), and social cohesion^63^, and complement with questions regarding place-making. Participants were exposed to an urban, peri-urban, and rural landscape represented by a multisensory combination of visual and auditory stimuli via 2D 360° virtual reality immersion. The study was conducted in the Netherlands and Switzerland with two sets of stimuli in each country to connect local observations to more widely applicable explanations of the role of emotions in place-making. While it is known that characteristic landscape elements can evoke strong feelings^64–67^, the fast growing peri-urban landscapes often lack typical ‘urban’ attributes (such as e.g. dense building structures, public spaces, or small retail shops), while also lacking the ‘naturalness’ associated to the ‘rural’ realm. We thus anticipate that homogenized peri-urban landscapes will trigger lower emotional responses, that will lead to lowering place-making. First, we demonstrate that both tonic and phasic signals are lower in peri-urban landscapes compared to the urban and rural landscapes. These results are consistent with the cognitive arousal and the valence results, showing significant lower excitement and pleasure in peri-urban areas. Finally, we show that place-making is well explained by cognitive and physiological emotional responses as well as social cohesion and place-attachment in urban and rural landscapes. In contrast, emotion is not significantly contributing to explaining place-making in the homogenized peri-urban landscapes.

**Figure 1:**
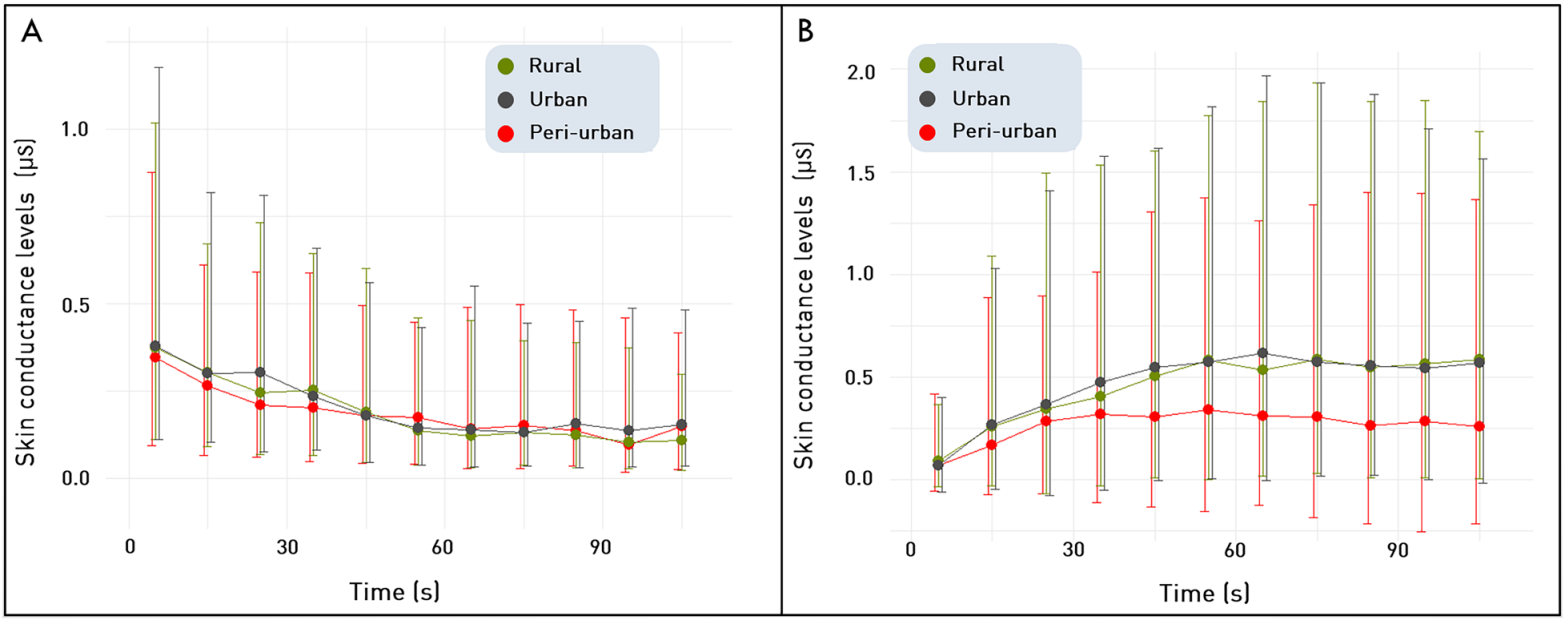
Physiological arousal measured by skin conductance responses (ΔSCL between baseline and stimulus) across the rural, urban and peri-urban scenes, with (**A**) phasic signal, and (**B**) tonic signal. Error bars represent the first and third quantiles of the dataset across 285 participants exposed to one of the two sets of stimuli from each type of residential landscapes (urban, peri-urban, and rural) in Switzerland and the Netherlands.

Firstly, participants perceived the peri-urban to be distinct from the urban and rural landscapes. They had significantly lower phasic and tonic signals when exposed to the peri-urban scenes than when they experienced the urban and rural scenes across the two sets of stimuli in each of the two countries (Fig. 1). The decay of the phasic signal of the participants that experienced the peri-urban landscapes was lower compared to the other stimuli. This is reflected in the significantly smaller slope angle of the phasic signal for the peri-urban stimuli (Friedman test, χ^2^ (2) = 10.2, p = 6.1e-3) and smaller areas under the curve of the phasic (Friedman test, χ^2^(2) = 16.4, p = 3e-4) and the tonic signal (Friedman test, χ^2^(2) = 8.9, p = 1.1e-2) compared to the rural and urban scenes (Supplementary Fig. S1). A Generalized Linear Mixed Model (GLMM) accounting for repeated measures confirmed the results of the Friedman test, showing significant changes in the physiological responses between the scenes with the lowest signals in the peri-urban scenes. Furthermore, as expected, the order of the presentation of the scenes had no effect on the results (Supplementary Table S1).

**Figure 2:**
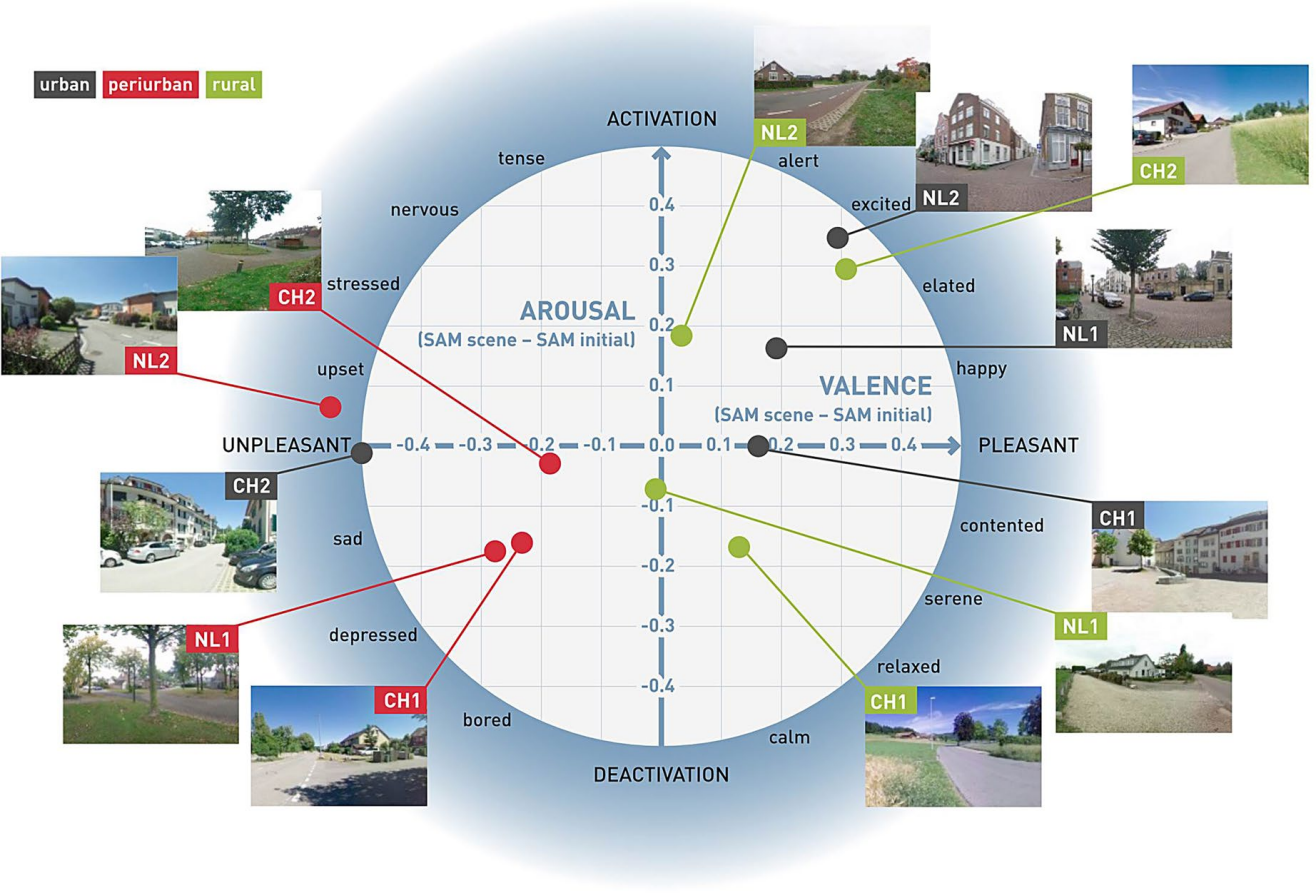
Representation of conscious arousal and valence assessments across participants into Feldman Barett and Russel circumplex model of affective reactions^64^. Arousal and valence measurements are means of the participants’ responses, and were adjusted with the initial status of each participant. The numbers 1 and 2 refer to the two scenes in Switzerland (CH), respectively the Netherlands (NL).

Secondly, conscious arousal and valence using the SAM pictograms confirmed the differences between the peri-urban scenes and both the rural and the urban scenes (Fig. 2). In particular, SAM valence showed overall negative assessments across the peri-urban scenes (Supplementary Fig. S2). Using the two-dimensional circumplex model of affective reactions of Feldman Barett & Russel^68^, we show that peri-urban scenes are consistently perceived unpleasant and trigger most of the time a low arousal across the participants and countries. In contrary, the rural scenes are mostly located in the pleasant quadrant with a marked difference between the countries, showing more pleasant reactions in Switzerland than in the Netherlands. The urban scenes also show important differences between countries. While the urban scenes from the Netherlands are all activating and pleasant, the Swiss scenes are neutral in arousal and one of the scenes is even unpleasant.

Finally, a regression model shows that place-making can be explained by affective factors in all the landscapes except in peri-urban areas (Table 1, and Supplementary Table S2 for the full model). Important to note is that the place-making questions were only asked for the scene which resembles most to the place the participants are currently living in, as a pre-test showed that people were only able to reliably answer such questions if they were acquainted to the places. In rural landscapes, we observe that place-making is best predicted by the phasic signal and social cohesion (*R*^*2*^*-adjusted* = 0.49, *p* = 4.3e-4), while in urban landscapes, valence comes along with factors related to social cohesion and place attachment (*R*^*2*^*-adjusted* = 0.22, *p* = 1e-6). In contrast, the peri-urban place-making is only weakly explained by place attachment with no influences of physical or stated arousal (*R*^*2*^*-adjusted* = 0.13, p = 2.7e-4). Similar results were generated when taking all the scenes together using a Generalized Linear Model (GLM), with the type of scene coded as categorical variable and including variable interactions with the type of scenes (Supplementary Table S3), but showing a lower model accuracy (*R*^*2*^*-adjusted* = 0.18, *p* = 4.67e-6).

**Table 1:** Relationships between place-making and emotion-related factors by type of scene.

	Rural scene coef	Urban scene coef	Peri-urban scene coef
(Intercept)	1.67***	1.83***	2.64***
Phasic area (physiological)	− 0.59**	–	–
Phasic slope (physiological)	− 0.42*	–	–
SAM valence (conscious)	–	− 0.20*	–
Social cohesion	0.64***	0.32**	–
Place attachment	–	0.25*	0.19*
*R*^*2*^*-adjusted*	*0.49*	*0.22*	*0.13*
*P-value*	< *0.05*	< *0.05*	< *0.05*
*N*	*41*	*127*	*103*

*p < 0.05, **p < 0.01 and ***p < 0.001.

Overall, the results are consistent with other studies showing that unconscious reactions to visual environmental contexts are important indicators for behavioral responses^18,69,70^. As observed by other authors, the unconscious arousal is, however, highly dependent on the degree of context involvement, in particular the familiarity to the place^18,71,72^. In rural areas, for example, the physiological reactions occur without extensive cognitive encoding, while participants in the urban areas expressed affective judgments, which were not related to the skin conductance level. Interestingly, the measured physiological responses are not correlated to the amount of green in the stimuli (Supplementary Table S4), and we even observed lower arousal values despite higher content of greenery, contrasting with results shown in other studies^24,38^. This might be due to a “misfit” between the amount of green and the meanings people ascribe to certain places. Individual judgments have been shown to overlap with the assignment of meaning and significance, and decisions about what people evaluate as “fitting” can drive people’s evaluations^73,74^. A better understanding of the perceived and revealed characteristics of the scenes, as recently investigated using novel approaches originating from the fields of machine learning and computer vision^75,76^, and their relationships to people’s emotions might provide cues on why the physiological responses to urban and rural areas are similar.

While our study is highly context-specific, we observe no significant differences between place-making across the two case studies conducted in two different countries (Wilcoxon test, *W* = 9190, *p* = 0.55). Furthermore, place-making was independent of gender, age, income, education, payed rent, occupation, number of people in the household, number of children in the household, length of residence, commuting time, recreation time, type of neighbourhood where they currently live, and type of neighbourhood where they grow up. Only housing situation (rent/ownership), the type of housing, the working time, and the type of neighbourhood where they lived most of their lives had a significant influence on the place-making scores (Supplementary Table S5). This confirms previous research showing that people who own their homes are likely to live there in the long term, which is related to place attachment and place identity^15,77,78^. In contrast, we do not observe relationships between place-making and length of residence^78,79^, income^80^, or gender^81,82^. As cities are characterised by pluralism of people and uses, future studies might want to systematically investigate personal differences and relationships to place-making across space and time to promote the inclusion of differences in cities^83,84^.

In summary, our results show that peri-urban landscapes trigger unpleasant and deactivating emotional responses compared to urban and rural landscapes. The unconscious physiological responses are supported by self-assessed measures, such as valence and conscious arousal. Relating these results to traditional measures of people–place relations shows that not only unconscious arousal influences place-making, but that social cohesion and place attachment are key factors explaining people’s engagement with place. These findings are in line with studies that have indicated the importance of the link between sense of place and intense caring for the locale^10,85,86^, but this study is the first to demonstrate the role of bodily responses in the intention to interact with places. In order for individuals to actively engage in place-making, both the affective and cognitive processes need thus to be considered by planners and policy makers^87,88^. Such relationships have relevant implications for fostering people’s engagement with places, and call for interventions in the ongoing homogenization process to steer urban transformation towards more liveable environments.

## Methods

### Participants

The study was conducted in two European urban areas, Utrecht (Netherland) and Olten-Aarau (Switzerland), both characterized by important population growth (0.5–1% per year) and strong residential pressure in peri-urban areas. A total of 285 participants (41.4% women, 57.2% men, mean age = 46, SD = 17.04, age range = 20–83 years) were recruited on the street using a mobile virtual reality lab installed in a van. The mobile lab allows testing audio-visual simulations in controlled conditions at any location that is reasonably quiet. The locations were changed every second to third day to recruit participants from different neighbourhoods. Participants were eligible for inclusion if they reported they were older than 18, physically and psychologically healthy, and with normal or corrected-to-normal vision and hearing. Participants were not directly compensated, but were allowed to participate in a lottery system for a price of 1500 Euros. The majority of participants (29.8%) had a higher specialized school education followed by master’s degree, doctoral research degree or advance studies (23.5%). Considering their occupations, 32.9% were fulltime workers, 22.4% part-time workers, and similar proportions between students (14.7%) and pensioned (16.1%). Most of the participants were locals with only a little proportion of foreigners (3.9%). The study was approved by ETH Zürich ethics commission (EK 2020-N-34), and performed in accordance with relevant guidelines and regulations. All participants provided written informed consent prior to the start of the experiment.

### Virtual reality setting

**Figure 3:**
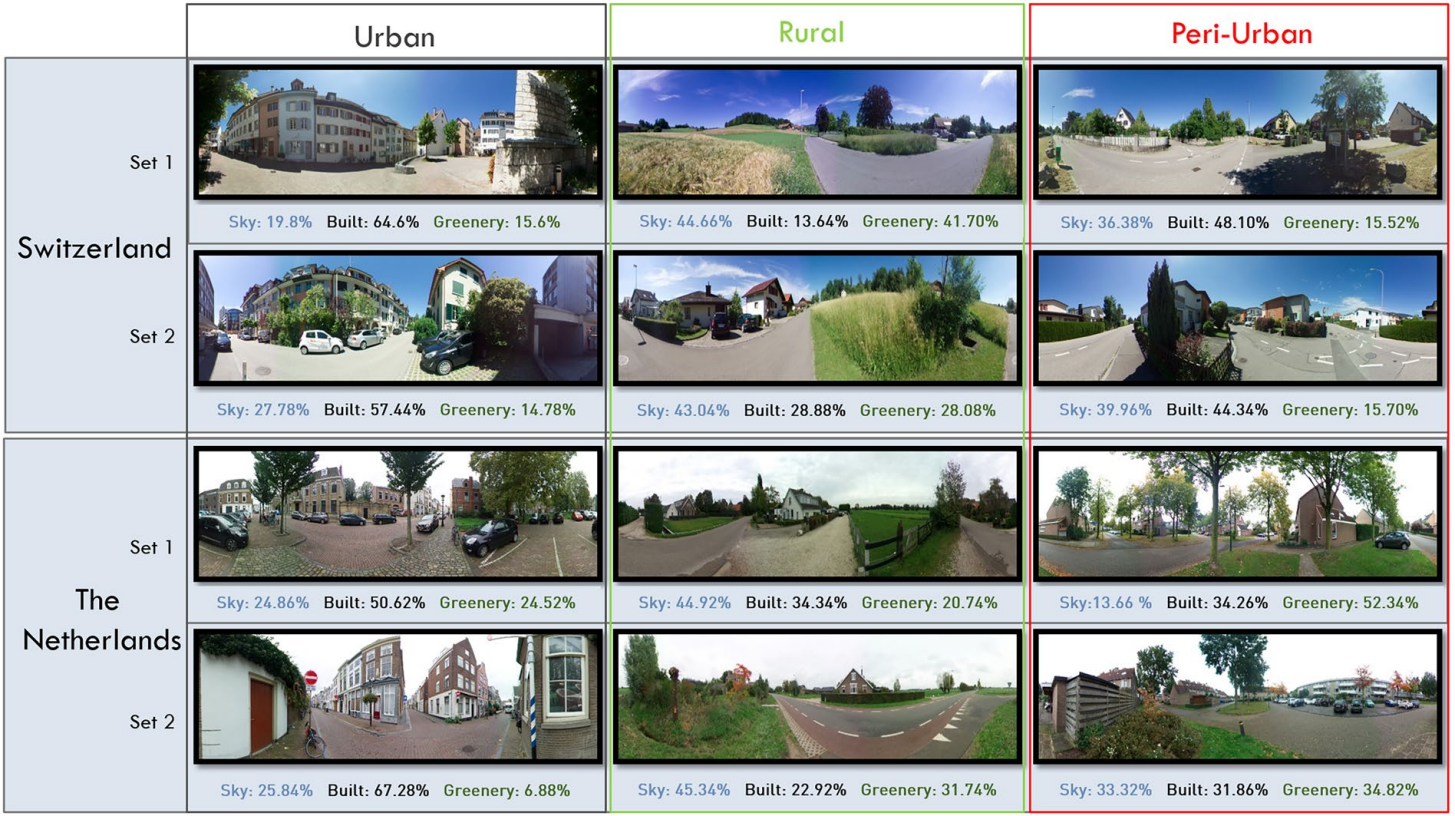
360° pictures of residential areas shown during the virtual reality experiment, including an estimation of the amount of sky, built, and greenery based on pixel count percentages. Images treated with Affinity Photo 1.10.0.1127 (https://affinity.serif.com).

The experiment included two sets of three 2D 360° Virtual Reality (VR) photos of an urban, a rural, and a peri-urban residential scene, respectively. The photos were taken using an Insta360 ONE X camera placed 2 m away from a street on a sidewalk or grass. Locations were selected based on relating socio-economic characteristics of a large online panel survey (N = 10′949 persons) (http://periviewer.ethz.ch) with urban characteristics, allowing to identify a gradient from urban to rural (Supplementary Fig. S3). Characteristics of each scene, including the share of sky, built environment, and green areas in the pictures, are provided in Fig. 3. Ambisonic sound was recorded with a Sennheiser AMBEO VR Mic at each location. Both the visual and auditory stimuli were pre-processed to generate characteristic stimuli of the locations. The photos were sharpened, people and moving cars were removed, and the light corrected with photo editing software. Locally recorded bird songs were mixed with traffic noise and calibrated to the sound levels at the various locations. In general, urban scenes were characterized by being louder with more traffic noise in the background. Both rural and peri-urban scenes were quieter with more bird singing sounds with occasional traffic noise. All scenes were acquired during the month of September in the morning to avoid pedestrians. The 360° images and the sound were combined in a VR setting in the game engine Unity (V. 2018.3.3 fl, www.unity.com).

### Experimental procedure

**Figure 4:**
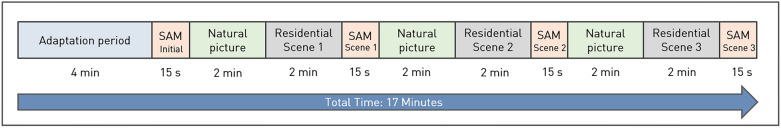
Overview of the experimental procedure using Virtual Reality stimuli in a mobile acoustic-visual lab. *SAM* Self-Assessment Manikin to measure valence and cognitive arousal. Skin conductance levels were measured along the entire experimental procedure. Total time changes depending on the time each participant used to grade the SAMs (15 s only as a reference).

The experiments were conducted in a mobile virtual lab installed in a van, specifically designed for visual-acoustic experiments, and tested in the AudioVisual lab (AV Lab) at ETH Zürich^89^. Pictures of the set-up in the field are provided in Fig. S4. Upon arrival, the respondents provided signed informed consent and were informed about the experiment. The experiment consisted of two parts, starting with the VR experiment, in which SCL were measured, followed by an extensive questionnaire. Participants were invited to sit in the van and were provided with a VR mask with integrated earphones^90^. The used HTC Vive headset has a dual RGB screen with low persistence LCD, a resolution of 2448*2448 pixels per eye (4896*2448 pixels combined), a field of view of 120°, and a refresh rate of 90/120 Hz. The experimenter then attached and calibrated the electrodermal electrodes^44^. The VR part started with a four-minutes adaptation period, during which participants read a children story presented in the VR googles, accompanied by Tibetan chants that are known to induce a relaxed but attentive state^91^. The purpose of this initial part was to make participants comfortable with the set up and the VR device on their heads. After the adaptation part, the participants were asked to self-assess their valence and arousal^55^. The experiment started with a two-minutes exposure to a natural scene accompanied by relaxing chants. The natural picture (tree canopy) was taken from the International Affective Picture System^92^ and is known to relax people. After being exposed for two minutes to the residential scenes, participants had again to self-assess their valence and arousal. The procedure was repeated for the three scenes (Fig. 4). In order to reduce people’s time commitment in the experiment, we limited the scene exposure to two minutes, which corresponds to a situation where both the tonic and the phasic signals reach stable conditions, based on pre-tests in the AV Lab. The order of the stimuli was randomized. After finishing the VR experiment, the experimenter removed the electrodes and participants were asked to complete the questionnaire outside the van. The experiment was deployed within EVE (Experiments in Virtual Environments), a comprehensive computing framework for the presentation of virtual environments and the collection of behavioural and physiological data^44,48^.

### Measures

#### Physiological measures

Skin conductance electrodes (MTL118F) from ADInstruments (https://www.adinstruments.com) were attached to the middle phalanges of the index and ring fingers of the non-dominant hand without pre-treatment of the skin. These electrodes were also attached to a FE116 GSR Amp and a Powerlab 8/35 recording device from ADInstruments. Data was collected at a sample rate of 1000 Hz using the software LabChart. Temperature and humidity could not be controlled inside the mobile lab, but all measurements were performed during the month of September with more or less stable weather conditions (Olten-Aarau (CH): average temperature 14.1 °C, relative humidity 77%, Utrecht (NL): average temperature 12.7 °C, relative humidity 75%) (www.world-weather.info). EDA measurements were first visually inspected for artefacts (low signals, interrupted signals, out of range signals, or no signals). Data with artefacts was discarded (18%). Several window times were tested to improve the estimation of the parameters of the impulse response function. The data was then decomposed using the Matlab-based software Ledalab (www.ledalab.de) into the average phasic and tonic activity using a continuous decomposition analysis (Benedek & Kaernbach, 2010) in 10 s-windows. The algorithm considers the response window starting 1 s after stimulus-onset to 4 s after stimulus-offset to consider the delay in sweat gland activation. The last 10 s-window of the 2 min-exposure were not used since it includes some initial seconds of the SAM grading part. Baseline skin conductance levels were obtained during the natural scene. Phasic signals were standardized using the minimal baseline skin conductance level, while tonic signals were standardized using the average last 10 s of the natural scenes. Because our experiment measures arousal in a continuous stimulus setting, we analysed differences between stimuli considering the EDA data as time series during the two-minutes exposure. Furthermore, because changes in these EDA parameters have a slow pace of change during short periods of time, we considered those time series to be linear between the beginning and the end of the two-minutes exposure, and we thus analysed the slope angles and the areas under the curve of both phasic and tonic signals ^93^ (Leiner et al. 2012).

#### Self-assessment measures

After each stimulus, participants were asked to rate their emotions using a controller that appeared in the VR as a laser pointing at the different SAM pictograms, measuring valence (from happy to unhappy) and arousal (from excited to calm). Each dimension is represented by images along a 5-point scale. After completing the physiological part of the experiment, participants filled out a questionnaire, which included questions related to place attachment, place-making and social cohesion. Socio-demographic information was also recorded including gender, age, nationality, income, education, rent, occupation, housing type, ownership, number of people in the household, number of children in the household, length of residence, amount of time spent in commuting, recreation time, working time and questions about the type of neighborhood where the participants grow up, currently live and where they have lived most of their life. Questions related to social cohesion were based on^63^, neighbourhood attachment on^94^, and place-making using the questionnaire proposed by. The latter questions are provided in the Supplementary Table S6. Items were rated on a five-point Likert scale from (1) strongly disagree, (2) disagree, (3) neutral, (4) agree, to (5) strongly agree. The questionnaire was set-up in a web application (www.soscisurvey.de), and displayed on a tablet device.

### Data analysis

To identify if there were differences in physiological reactions between the urban, rural, and peri-urban scenes, we utilized the Friedman non-parametric test^95^, given violations of assumptions for normal distribution (Supplementary Table S7). Friedman tests showing differences between the three stimuli were followed by pairwise comparisons using Wilcoxon tests with Bonferroni adjustment. Furthermore, to account for repeated measures and to test for the order effect of the scenes, we used a Generalized Mixed Method Model (GLMM) (with lmer() function from R package lme4)^96^, robust against violations of distribution assumptions^97^.

We explored the relationships between place-making, place attachment, social cohesion and affective reactions using a GLM presented in Table S3. As the place-related questions were only asked for the place the participants were living in, we could not use a GLMM but integrated the scenes as categorical variable in the GLM. We also present three separate regressions splitting our sample by considering where the participants are currently living in Table 1. Among the 285 participants, 127 of them declared to be actually living in an area similar to the urban scene, 103 in an area similar to the peri-urban scene and 41 in an area similar to the rural scene. The rest (14) declared that they currently live in an area that does not look similar to any of the three proposed scenes. After cleaning for incomplete answers in the survey, the sample got reduce to 113 participants for urban, 91 participants for peri-urban and 33 for rural areas. For each group, we performed a regression analysis using a stepwise regression method^98^, where the dependent variable was place-making, and independent variables were social cohesion, place attachment and the self-reported SAM valence, SAM arousal, as well as the physiological measurements of emotion triggered by the physical environment, which included the phasic slope, the phasic area under the curve, the phasic intercept, the tonic slope, the tonic area under the curve, and the tonic intercept.

We first performed a correlation analysis using Spearman’s correlation^99^ to discard correlated independent variables from the models (Supplementary Fig. S5). We then used the Akaike information criterion (AIC) in a stepwise procedure in order to reduce the models to only the significant variables (with the stepAIC() function from the R package MASS)^100^. We standardized the regression coefficients (with the lm.beta() function from R package QuantPsyc)^101^ to be able to compare them. Results presented in Table 1 are in accordance with significance levels found in the complete model provided in the Supplementary Table S3, and with the GLM presented Table S3. All the tests and regression analyses were conducted using R-Studio (version 1.3.1093).

## Supplementary Information

Supplementary Information.

## Data Availability

All data generated or analyzed during this study are included in this published article (and its Supplementary data).
