# Supplementary material for: Global urban homogenization and the loss of emotions
Source: Sci Rep. 2022 Dec 29;12:22515. doi: 10.1038/s41598-022-27141-7 (PMC9800410; doi:10.1038/s41598-022-27141-7)
Supplement: Supplementary file 1 — Supplementary Information. [file 41598_2022_27141_MOESM1_ESM.pdf]

Global urban homogenization and the loss of emotions

Supplementary data

MAIN

(1) Figure S1 – Differences of phasic and tonic signals between urban, peri-urban, and rural scenes

(2) Table S1 – Generalized Linear Mixed Model (GLMM)

(3) Figure S2 – SAM valence across residential scenes.

(4) Table S2 – Regression analysis of place-making to emotion-related factors by type of scene

(5) Table S3 – General Linear Model (GLM) of place-making to emotion-related factors

(6) Table S4 - Relationships between greenery and physiological measurements

(7) Table S5 – Influence of socio-demographic characteristics of participants on place-making

METHODS

(1) Figure S3 – Study sites

(2) Table S6 – Place-making questions

(3) Table S7 – Distribution of physiological measurement values

(4) Figure S4 – Pictures of the experimental set-up in the field

(5) Figure S5 – Correlations matrices of variables used in the regression analysis

MAIN

Figure S1 – Differences of phasic and tonic signals between urban, peri-urban, and rural scenes

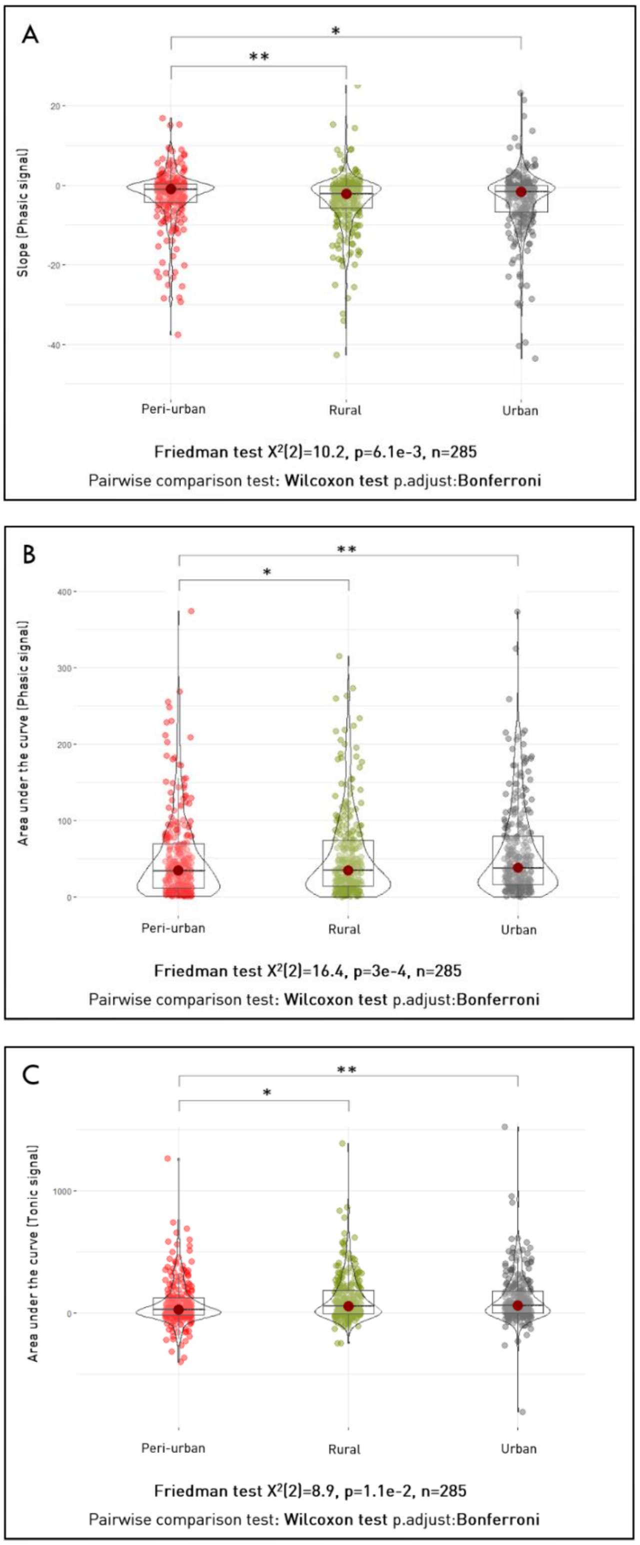

Figure S1: Analysis of differences of slope angle of the phasic signal (A), area under the curve of the phasic signal (B) and area under the curve of the tonic signal (C) between three types of residential scenes (peri-urban, rural and urban) represented each by two stimuli in both Switzerland and the Netherland. A Generalized Linear Mixed Model (GLMM) analysis shows the same results, except for the case of the area under the curve of the phasic signal that shows differences between the rural-urban scenes. Asterisks show adjusted p-value significance.

Table S1 – Generalized Linear Mixed Model (GLMM)

Table S1: Generalized Linear Mixed Model (GLMM) to investigate the role of order of the scene on the physiological measurements.

	Model[1]
Slope Angle	Scene, *F = 4.7, p=0.009**<br>Order, *F = 1.4, p=0.235*
Phasic area under the curve	Scene, *F = 3.6, p=0.028**<br>Order, *F = 0.8, p=0.967*
Tonic area under the curve	Scene *F = 5.2, p=0.005**<br>Order, *F = 0.5, p=0.786*

[1] Random effect of participants with fixed effect of the order of the scenes

Figure S2 – SAM valence across residential scenes

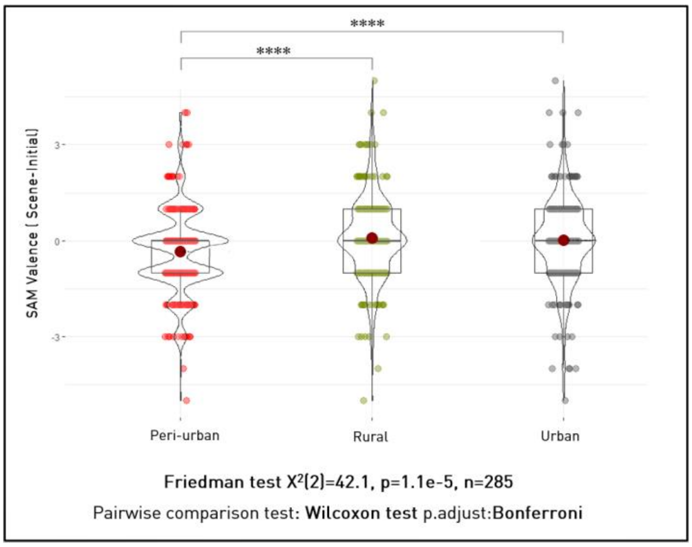

Figure S2: Differences of valence measurements with the self-assessment manikins (SAM) between types of residential scenes (peri-urban, rural and urban). Asterisks show adjusted p-value significance.

Table S2 – Regression analysis of place-making to emotion-related factors by type of scene

Table S2: Regression analyses of place-making to emotion-related factors by type of scene, including the full and the reduced models.

	Rural (n=33)		Urban (n=113)		Peri-Urban (n=91)	
	Full model	Reduced	Full model	Reduced	Full model	Reduced
(Intercept)	1.66***	1.67***	1.85***	1.83***	2.46***	2.64***
Phasic area	-3.9e-2**	-0.59**	9e-3	-	-1.1e-3	-
Phasic slope	-27.8*	-0.42*	-2.31	-	6.52	-
Tonic area	-4.4e-3	-	-4e-3	-	-8.4e-4	-
Tonic slope	5.14	-	-1.3	-	-1.16	-
SAM valence	1.1e-2	-	-7.7e-2*	-0.20*	-7.6e-3	-
SAM arousal	-6.1e-2	-	1.6e-3	-	-4.4e-3	-
Social cohesion	0.52***	0.64***	0.27**	0.32**	0.1	-
Place attachment	-3.7e-2	-	0.15*	0.25*	0.14*	0.19*
R^2-Adjusted	*0.43*	*0.49*	*0.20*	*0.22*	*9.2e-2*	*0.13*
p-value	*< 0.05*	*< 0.05*	*< 0.05*	*< 0.05*	*<0.05*	*< 0.05*

* p<0.05, ** p<0.01 and *** p<0.001.

Table S3 – Generalized Linear Model (GLM) of place-making to emotion-related factors

Table S3: Generalized Linear Model of place-making to emotion-related factors by type of scene, including the full and the reduced models (n=237).

	Full model	Reduced
(Intercept)	2.03***	2***
Phasic slope interaction Peri-Urban	3.62	-
Phasic slope interaction Rural	-2.62e1	-
Phasic slope interaction Urban	-2.32	-
Phasic area interaction Peri-Urban	1.97e-3	-1.9e-3
Phasic area interaction Rural	**-4.11e-2***	**-2.4e-2°**
Phasic area interaction Urban	8.49e-3	2.6e--3
Tonic slope interaction Peri-Urban	-4.92e-1	-
Tonic slope interaction Rural	4.55	-
Tonic slope interaction Urban	-1.37	-
Tonic area interaction Peri-Urban	-8.74e-4	-
Tonic area interaction Rural	-3.95e-3	-
Tonic area interaction Urban	-4.19e-3	-
SAM valence interaction Peri-Urban	-2.9e-3	-7.4e-4
SAM valence interaction Rural	1.55e-2	-2.1e-2
SAM valence interaction Urban	**-7.39e-2***	**-6.9e-2***
SAM arousal interaction Peri-Urban	4.53e-3	-
SAM arousal interaction Rural	-5.91e-2	-
SAM arousal interaction Urban	4.44e-3	-
Social cohesion interaction Peri-Urban	**2.11e-1****	**2.2e-1****
Social cohesion interaction Rural	**4.52e-1*****	**4.3e-1*****
Social cohesion interaction Urban	**2.25e-1****	**2.3e-1*****
Place attachment interaction Peri-Urban	**1.52e-1***	**1.5e-1***
Place attachment interaction Rural	-6.53e-2	-3.6e-2
Place attachment interaction Urban	**1.42e-1***	**1.5e-1***
R^2-Adjusted	*0.18*	*0.20*
p-value	*<0.05*	*<0.05*

* p<0.05, ** p<0.01, *** p<0.001, and ° p<0.1.

Table S4 – Relationships between greenery and physiological measurements

Table S4: Spearman's correlation between greenery and physiological measures. The analysis is based on an average value for each scene.

	Spearman's correlation (**ρ**)	p-value
Phasic slope	-0.09	0.78
Phasic intercept	-0.23	0.47
Phasic area under the curve	-0.26	0.42
Tonic slope	-0.44	0.15
Tonic intercept	0.20	0.53
Tonic area under the curve	0.29	0.37

Table S5 – Influence of socio-demographic characteristics of participants on place-making

Table S5: Correlations between place-making scores and participants' socio-demographic characteristics. Origin (local/foreigner) could not be tested because the foreigner group was too small (only 3.9% foreigners). Asterisks indicate the level of significance of the p-value. H refers to the results of the Kruskal-Wallis test, while ρ corresponds to Spearman's correlation.

Factors	Test	p-value
Gender	H(3)=0.71	0.87
Age	ρ =0.14	0.11
Income	H(5)=3.9,	0.55
Education	H(7)=13.8	5.4e-2
Rent	ρ =-5.8e-2	0.56
Occupation	H(6)=11.9	6.2e-2
Type of housing	**H(3)=11.4**	9.9e-3**
Housing ownership	**H(3)=15.9**	1.2e-3**
Nr. people in household	ρ =0.03	0.73
Nr. children in household	ρ =0.06	0.46
Length of residence	ρ =0.12	0.21
Commuting time	H(5)=8.8	0.11
Recreation time in neighbourhood	H(5)=10.4	6.4e-2
Recreation time outside neighbourhood	H(5)=4.9	0.42
Working time	**H(6)=14.8**	2.1e-2*
Where lived most of life	**H(3)=13.6**	3.5e-3**
Where currently live	H(3)=1.9	0.58
Where lived growing up	H(3)=2.7	0.44

METHODS

Figure S3 – Study sites

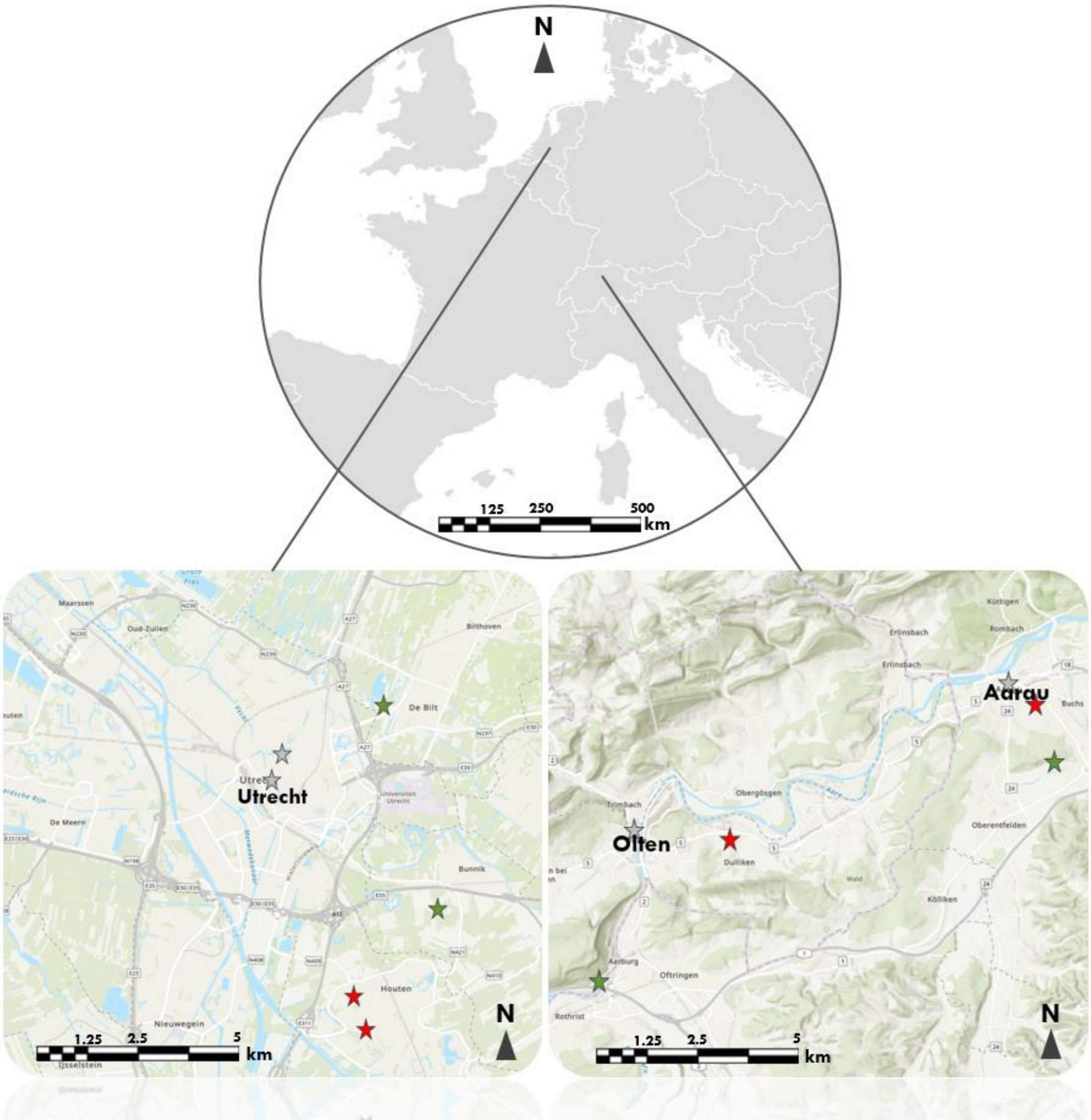

Figure S3: Location of the residential scenes showed during the experiment. Grey stars show the locations of urban scenes, green stars show the locations of rural scenes and red stars show the locations of peri-urban scenes. Map generated using ArcGIS Pro 2.6.0 (http://pro.arcgis.com).

Table S6 − Place-making questions

Table S6: Place-making questions based on Switalski et al. (in revision)[64].

Concept	Item
Design	The design of my neighborhood needs to be changed.
Nature	There is plenty of nature which enriches my neighborhood.
Density	In my neighborhood, we are living too close together.
Participation	I would like to participate more in how my neighborhood changes.
Upkeep	I would like to support the upkeep of my neighborhood.
Walkability	My neighborhood is a place where I can participate in improving the walkability.
Initiatives	I support initiatives which influence how my neighborhood changes.
Furniture	I am happy with the possibilities to influence the street furniture in my neighborhood.
Leisure	My neighborhood is a good place for leisure activities.
Recreation	It is possible to change how people recreate in my neighborhood.
Activities	The type of activities in the public spaces in my neighborhood could be changed.
Interactions	It is possible to change the types of social interactions in my neighborhood.
Safety	I would like to help make my neighborhood feel safer or livelier.
Home	My neighborhood is a place where people can feel at home.

The survey questions were designed to cover the variety of ways in which places can be understood, while having to also represent the inherently dynamic nature of place-making. Literature spanning a large thematic breadth was used, ranging from urbanism and architecture to environmental psychology or sociology.

Table S7 − Distribution of physiological measurement values

Table S7: Distribution of physiological measurement values. Shapiro-Wilk normality tests on the variables.

	Shapiro-Wilk Test (W)	p-value
Peri-urban phasic slope	0.52	<2.2e-16
Urban phasic slope	0.62	<2.2e-16
Rural phasic slope	0.76	<2.2e-16
Peri-urban phasic area under the curve	0.68	<2.2e-16
Urban phasic area under the curve	0.64	<2.2e-16
Rural phasic area under the curve	0.80	<2.2e-16
Peri-urban tonic slope	0.64	<2.2e-16
Urban tonic slope	0.43	<2.2e-16
Rural tonic slope	0.81	<2.2e-16
Peri-urban tonic area under the curve	0.85	<2.2e-16
Urban tonic area under the curve	0.81	<2.2e-16
Rural tonic area under the curve	0.82	<2.2e-16

Figure S4 – Pictures of the experimental set-up in the field

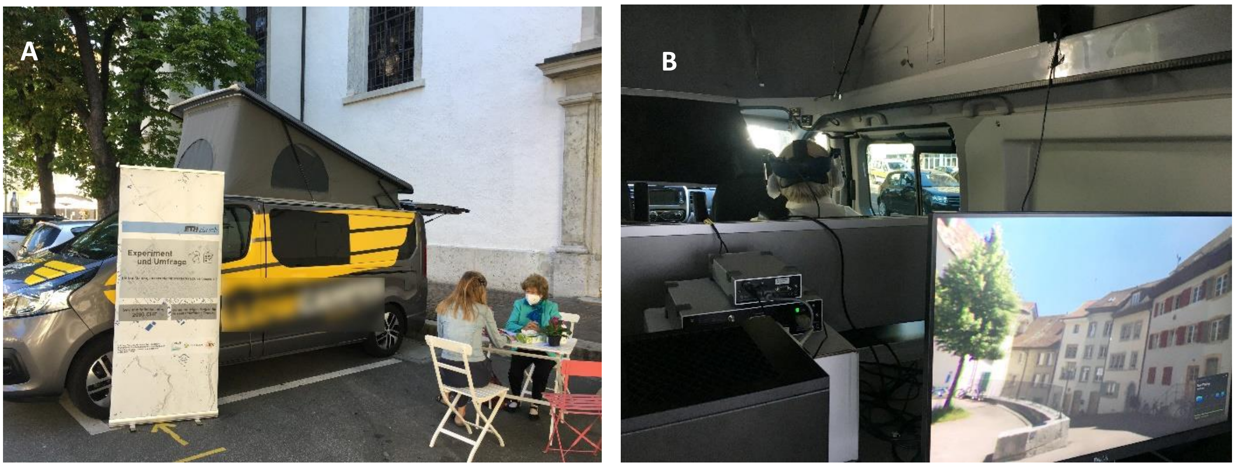

Figure S4: Pictures of the experimental set-up in the field, with a mobile lab in the van (A), and view into the inside of the van with the virtual reality instruments, including the computer installed in the back of the van showing what the participant currently sees in the VR googles (B).

Figure S5 – Correlations matrices of variables used in the regression analysis

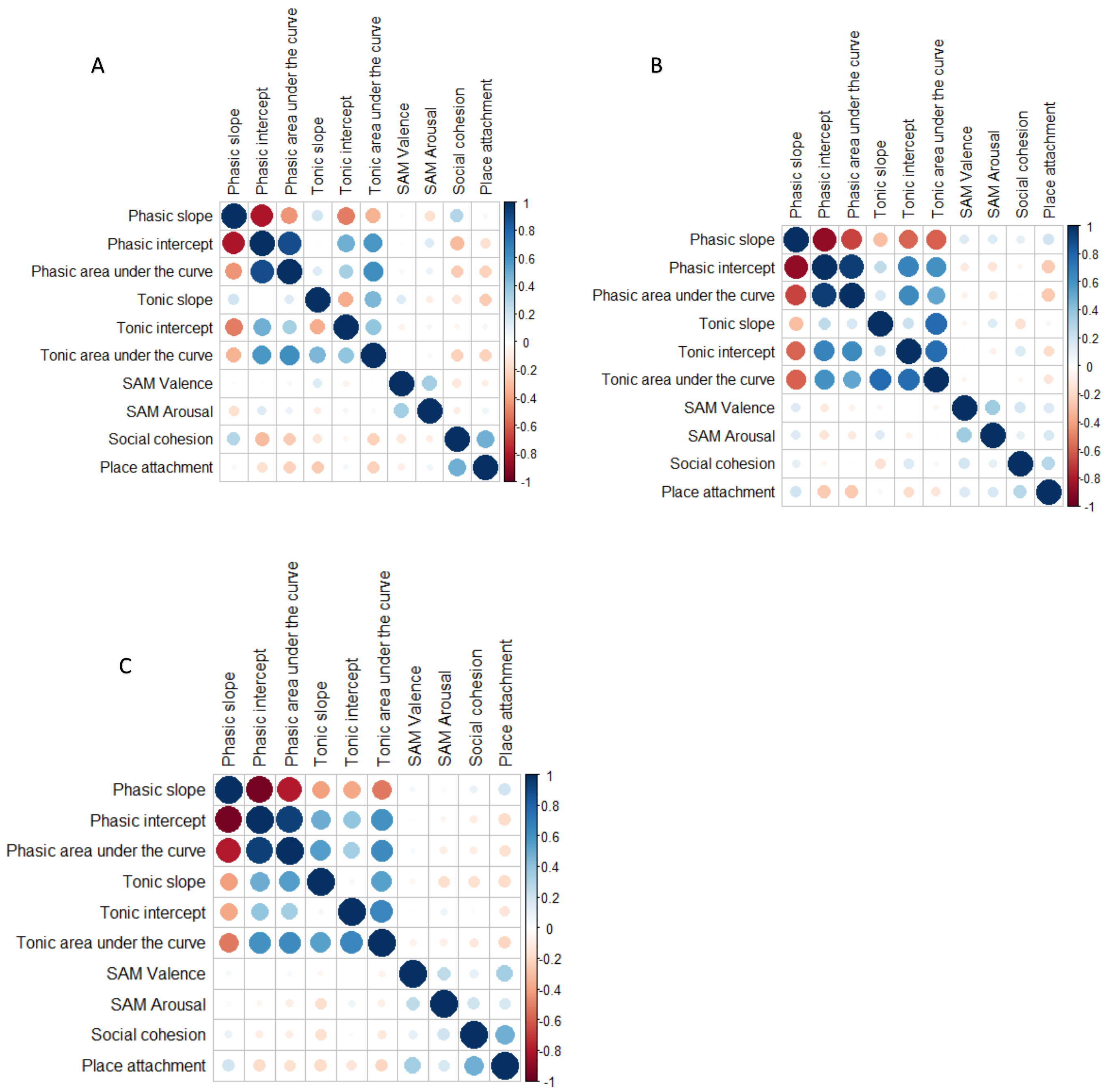

Figure S5: Correlation matrices (Spearman's correlation) of the variables used in the regression analysis. (A) Participants living in peri-urban areas, (B) participants living in rural areas, (c) participants living in urban areas. The phasic intercept variable was not considered in the regression, because of a high correlation with phasic slope and the phasic area under the curve.
